# The Possible Coupling of LNG Regasification Process with the TSA Method of Oxygen Separation from Atmospheric Air

**DOI:** 10.3390/e23030350

**Published:** 2021-03-15

**Authors:** Tomasz Banaszkiewicz

**Affiliations:** Department of Cryogenics and Aerospace Engineering, Wroclaw University of Science and Technology, Wybrzeze Wyspianskiego 27, 50-370 Wroclaw, Poland; tomasz.banaszkiewicz@pwr.edu.pl

**Keywords:** oxygen, adsorption, separation, energy engineering, LNG, regasification

## Abstract

Liquefied Natural Gas (LNG) must be vaporized before it is used in the combustion process. In most regasification terminals, energy that was previously expended to liquefy natural gas is dissipated in the environment. The paper proposes the use of the thermal effect of LNG regasification for the atmospheric air separation as a possible solution to the LNG exergy recovery problem. The presented idea is based on the coupling of the LNG regasification unit with an oxygen generator based on the Temperature Swing Adsorption (TSA) process. Theoretical analysis has revealed that it is thermodynamically justified to use the LNG enthalpy of vaporization for cooling of the TSA adsorption bed for increasing its adsorptive capacity. It has been shown that 1 kg of LNG carries enough exergy for separating up to approximately 100 g of oxygen using the TSA method. Although the paper suggests using the enthalpy of LNG vaporization for atmospheric air separation, similar processes for other gas mixture separations using the TSA method can be applied.

## 1. Introduction

Natural gas is one of the main natural energy sources, along with coal and oil. Its global use is increasing year to year. In 2017, it was, on average, a 3% increase globally and a 5.5% increase in Europe compared to the previous year [1]. Natural gas is considered the cleanest natural fuel. After natural gas combustion, the $CO_{2}$ emissions are 30% lower than from oil combustion and as much as 60% lower than when using coal [2]. The emission of $SO_{2}$, dust and aromatic hydrocarbons are also significantly lower. Natural gas is predominantly used as a source of energy in industry, transport and for the municipal needs in form of Liquefied Natural Gas (LNG) as it is more efficient to transport. Accordingly to the growing interest in LNG, it is advantageous to attempt to more comprehensively exploit this source of energy. Before natural gas is used, it needs to be gasified and pressurize to cope with the needs of the customer. The overall efficiency of the LNG utilization can be significantly improved with the change of currently used regasification processes. There are several methods of natural gas regasification. The process requires providing to the system an external heat equal to the sum of the enthalpy of natural gas vaporization and the heat needed to warm up natural gas to a desirable temperature. This is done, for example, by burning part of regasified natural gas, by use of electric heaters or by heat contact between LNG and ambient air or water (oceanic or river) [3]. In such cases, cooling power that was used to liquefy natural gas in the first place is wasted [4]. Although the total energetic cost of natural gas liquefaction is relatively low and does not exceed 0.031–0.102 $GJ/GJ_{LNG}$ depending on the scale [5], it is reasonable to consider technology of energy recovery during the regasification process.

In the standard tanks [6], and in the large scale tanks [7], the LNG is stored at the temp of 110 K. To evaporate and reheat to the ambient temperature, natural gas consumes about 1000 kJ/kg of heat. This heat flow, with the appropriate heat exchanger design, can be converted into a useful effect. The simplest way to recover that cooling power is to use it to cool down some refrigeration chambers [8], but it implies the necessity of building the refrigeration chambers in the near proximity of the LNG regasification terminal. The more probable and the most discussed in the literature is coupling the LNG regasification terminal with the dedicated thermodynamic cycle for electric power generation. The LNG phase change, theoretically, can be used as the low temperature heat source for ORC cycle to obtain up to 1.7 GWh electricity annually from 1 kg/s of LNG regasification [9]. Similar considerations were made for small LNG regasification rates below 0.2 kg/s [10]. In that case, the theoretical exergetic efficiency does not exceed 20%. The LNG exergy can also be used to increase the efficiency of the power cycle integrated with natural gas combustion [11].

Although the literature mainly focuses on the LNG energy recovery in the electric power generation, the LNG exergy can be used in different processes. The author of the paper shows that it is possible to use the low-temperature from the LNG–NG transition for the air separation using the Temperature Swing Adsorption (TSA) method. As the demand for the oxygen is increasing in the industry and especially in health care institutions, it would be reasonable to consider the use of the LNG cooling power to separate the oxygen from the air using TSA technology.

The natural gas composition varies depending on the place of its extraction [12,13]. Table 1 shows the natural gas composition for several chosen sources.

Due to the high variability of the composition, it is not possible to accurately calculate any thermodynamic variables that accurately reflect the thermodynamics of the natural gas. Table 1 shows that natural gas consists of a mixture of hydrocarbons, the main component of which is methane (70%–97%). For this reason, the author, for the sake of simplification, performs all calculations of the thermodynamics of natural gas using methane. The natural gas and methane are used alternatively in this paper. For typical natural gas used in Poland, the heat of vaporization varies between 502–508 kJ/kg [14]. The heat of vaporization for methane is approximately equal to 510 kJ/kg [14]. The error made by the simplification is small and will not significantly affect the calculations. The accurate calculations will be performed in the future with the experiment of LNG–TSA coupling. All the calculations in this paper were performed with the assumptions of no energy loss through the thermal isolation.

## 2. TSA Method of Air Separation

**Figure 1:**
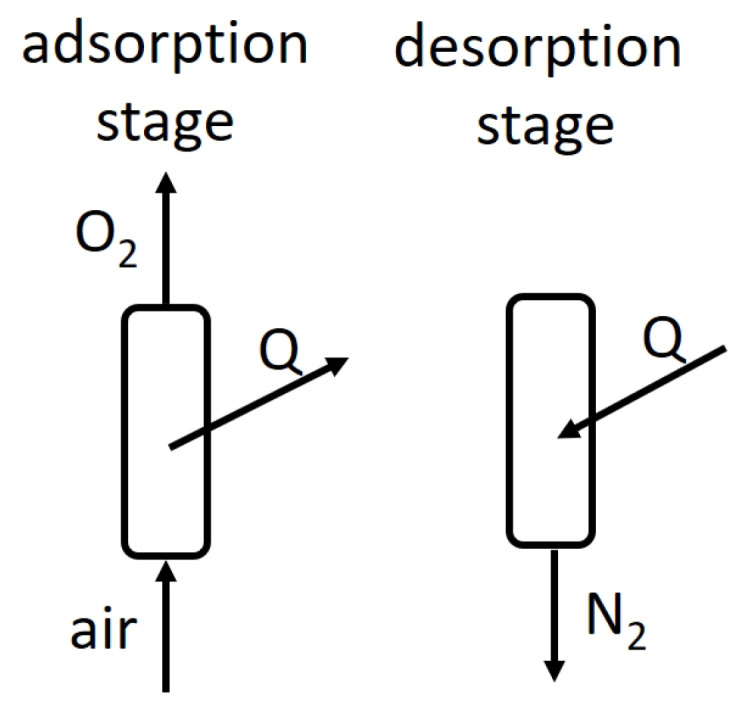
The scheme of the Temperature Swing Adsorption (TSA) method of air separation.

Adsorption is the process of accumulating gas-phase particles on the surface of a solid. The maximum capacity of the bed depends on the used adsorbent solid and adsorbed gas. Also, the amount of adsorbed particles strongly depends on temperature and pressure. The adsorption bed has a higher capacity for gas with a lower temperature and higher pressure [15]. The adsorption beds for gas mixtures separation are specifically selected so that the adsorption rate of one gas mixture component is several times greater than for the other. For oxygen separation from the air, usually, the nitrogen-selective beds, like zeolites 5A or 13X are used [16]. The Temperature Swing Adsorption (TSA) method uses the variable capacity of an adsorption bed at different temperatures and is driven at a constant pressure. The adsorption stage is performed in lower temperatures. In this stage, all the nitrogen gas from a fresh, dry portion of air is adsorbed on the adsorption bed. The remaining gas (composition: 95% oxygen and 5% argon) flows into the tank as the product. After the adsorption stage, the temperature of the adsorption bed is increased for the desorption stage. In this stage, the nitrogen gas is desorbed (released) from the adsorption bed and removed to the atmosphere. Figure 1 shows the basic scheme of the TSA method of air separation using the adsorption–desorption cycle.

**Figure 2:**
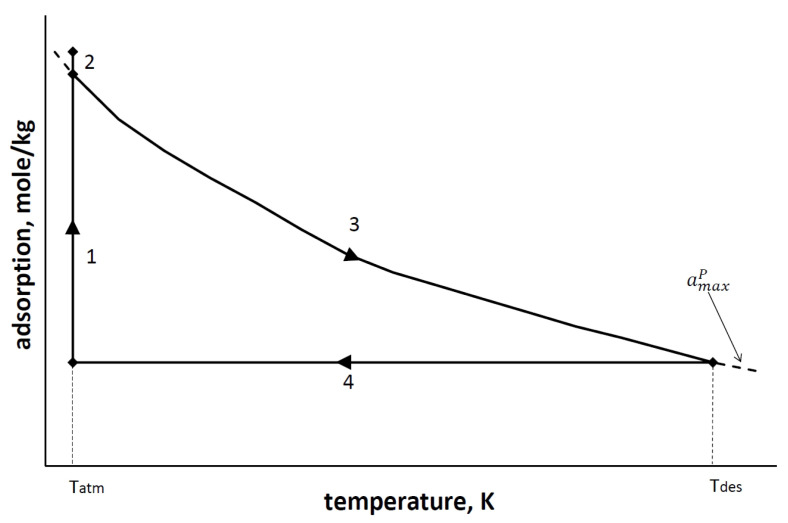
Adsorption cycle of the TSA method. $a_{max}^{P}$—maximum degree of adsorption at constant pressure, $T_{atm}$—atmospheric temperature, $T_{des}$—temperature of desorption stage.

In a standard TSA approach, the whole process occurs at atmospheric pressure, the adsorption stage takes place at ambient temperature, and the regeneration at increased temperature—usually at a temperature between 370–450 K [17]. Thus the temperature difference between the desorption and the adsorption stage usually varies between 70 and 150 K. This requires a supply of a significant amount of heat at this temperature level. Thanks to the natural gas regasification process, it is possible to move the TSA process to the lower temperature level. To preserve the temperature difference, the adsorption stage may be performed, for example, at the temperature of 200 K and desorption at an ambient temperature. The full cycle of the adsorption oxygen generator is called a prescription. The length and complexity of the adsorption cycle depend on the used technology, the type of sorbent and the expected quality of the product. Figure 2 shows the proposed, simple TSA cycle for the atmospheric oxygen separation.

**Figure 3:**
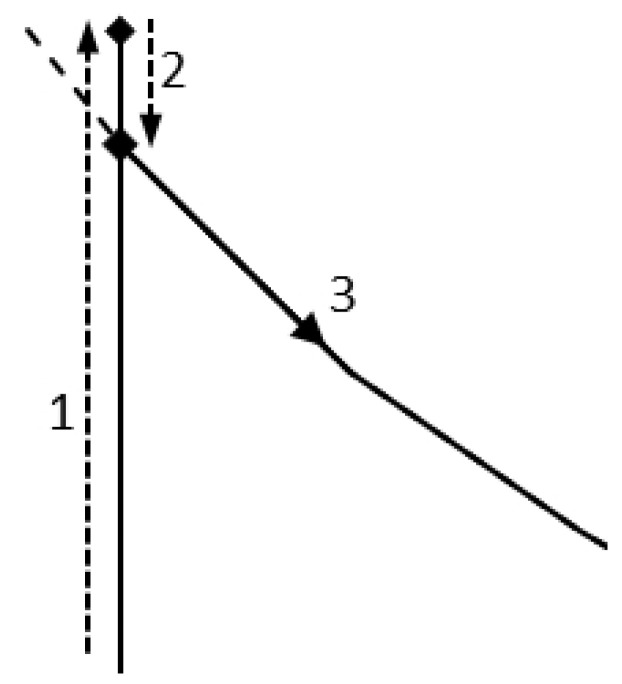
Enlargement of Figure 2.

The basic prescription for oxygen separation in cold variable temperature technology (TSA) consists of four stages:

1. Heat transfer—the main adsorption process. At this stage, the feed stream (clean air) enters the adsorber, which is cooled down to the temperature of liquid methane in the last phase of the previous cycle. Immediately the nitrogen gas is being adsorbed and the product stream (oxygen) is withdrawn. This process continues until the bed is saturated with nitrogen gas. During this stage, there is a slight increase in pressure in the adsorption tanks above atmospheric pressure. This increase is associated with the need for the gas to overcome the flow resistance along the adsorption bed. Increased pressure is also necessary to store the separated oxygen in the oxygen vessel. This results in a higher degree of adsorption than possible under atmospheric pressure (line 1 on Figure 2 and Figure 3).
2. Blow-out—this is the stage during which the pressure inside the adsorber is equalized with atmospheric pressure.
3. Regeneration-heating—the main desorption process. At this stage, the bed is reheated to the ambient temperature. The increased temperature of the adsorption bed reduces its adsorption capacity, causing the release of adsorbed nitrogen to the atmosphere.
4. Regeneration-cooling—immediately after the heating phase, the bed is cooled down using the cooling power of the regasified LNG. This stage lasts until the bed reaches the temperature needed in Stage 1.

Taking into account that dry air is a mixture of mostly two components—nitrogen and oxygen, it can be treated as the binary mixture with the $N_{2}/O_{2}$ composition of 78%/22%. The remaining air components, such as argon, will be treated as pollutants of the produced oxygen. To describe isotherms of oxygen and nitrogen adsorption from the air, the DSL model (Dual-Site Langmuir) for an oxygen–nitrogen binary mixture was applied [18].

(1) $$a_{O_{2}}=a_{md}\frac{K_{d-O_{2}}Py_{O_{2}}}{1+K_{d-O_{2}}Py_{O_{2}}+K_{d-N_{2}}Py_{N_{2}}}+a_{mb}\frac{K_{b-O_{2}}Py_{O_{2}}}{1+K_{b-O_{2}}Py_{O_{2}}+K_{b-N_{2}}Py_{N_{2}}}$$

(2) $$a_{N_{2}}=a_{md}\frac{K_{d-N_{2}}Py_{O_{2}}}{1+K_{d-O_{2}}Py_{O_{2}}+K_{d-N_{2}}Py_{N_{2}}}+a_{mb}\frac{K_{b-N_{2}}Py_{O_{2}}}{1+K_{b-O_{2}}Py_{O_{2}}+K_{b-N_{2}}Py_{N_{2}}}$$

where $a_{i}$ is the adsorption bed capacity for the *i*-th gas at the adsorption conditions, $a_{md}$$a_{mb}$ are the maximum adsorption bed capacity, $K_{b-i}$ and $K_{d-i}$ are the two affinity parameters of *i*-th gas the adsorption conditions, *P* is the adsorption pressure and the $y_{i}$ is the initial composition of the *i*-th gas.

For DSL isotherm, it is considered that the temperature dependence of $K_{d}$ and $K_{b}$ constants is consistent with the Arrhenius equation:

(3) $$K_{b-i}=K_{b-i}^{0}e^{\frac{Q_{b-i}}{R}\left(\frac{1}{T}-\frac{1}{T_{0}}\right)}$$

(4) $$K_{d-i}=K_{d-i}^{0}e^{\frac{Q_{d-i}}{R}\left(\frac{1}{T}-\frac{1}{T_{0}}\right)}$$

The $K_{b-i}^{0}$ and $K_{d-i}^{0}$ are the two affinity parameters of *i*-th gas at the reference temperature of $T_{0}$ and $Q_{b-i}$ and $Q_{d-i}$ are the heats of adsorption of *i*-th gas on the two sites. *T* is the temperature in which the adsorption process is conducted and the *R* is the gas constant.

Nitrogen adsorption was calculated with assumption of the Zeolite 5A adsorption bed usage. For good nitrogen and oxygen adsorption representation the constant values shown in Table 2 were used. Parameters shown in Table 2 were composed based on experimental data [19] showed in Table 3 and Table 4.

Equilibrium constants $K_{b}^{0}$ and $K_{d}^{0}$ were determined for temperature $T_{0}$ = 23 °C.

**Figure 4:**
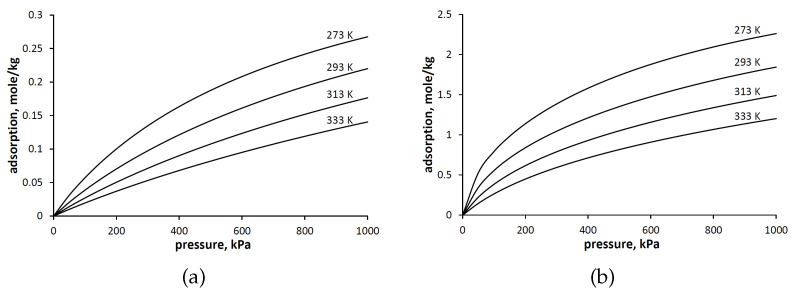
Oxygen (**a**) and nitrogen (**b**) adsorption isotherms on zeolites 5A for the different pressures.

Figure 4 shows nitrogen and oxygen adsorption isotherms on zeolites 5A that were calculated using Equations (2) and (3).

## 3. Optimal Temperature for TSA Oxygen Separation

The adsorption method of air separation is dependent on both adsorption and desorption conditions—pressure and temperature. In order to minimize the cost of the separation process, the method described in the paper was performed at a constant, atmospheric pressure. The desorption stage was performed at the ambient temperature whilst the temperature of the adsorption stage was reduced by the heat contact with the regasified natural gas.

**Figure 5:**
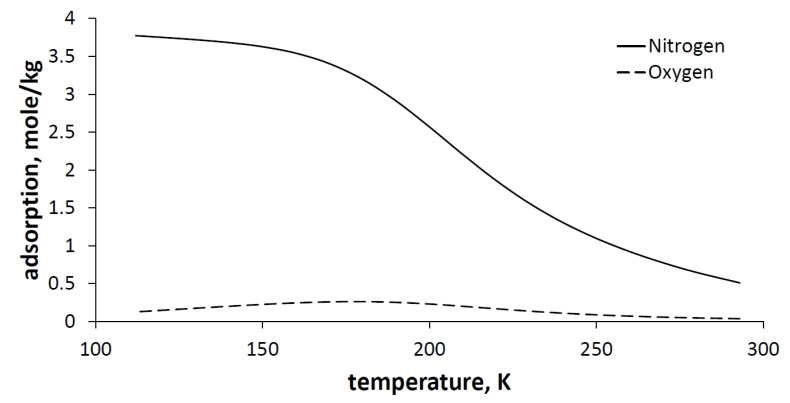
Nitrogen and oxygen adsorption isobars on zeolites 5A for the pressure 1 bar.

Since the pressure will be kept constant throughout the adsorption and desorption process, it is more useful to use an adsorption isobar instead of an adsorption isotherm. The nitrogen and oxygen adsorption isobars are shown in Figure 5.

At the adsorption stage, the fresh portion of the air is introduced to the adsorption bed. Then the molecules of nitrogen and, in a smaller amount, oxygen are removed from the air by the adsorption bed. During the desorption stage, the molecules are released from the adsorption bed to the atmosphere. The amount of molecules separated can be calculated as the difference between the molecules amount trapped in the adsorption bed in the adsorption and desorption stages:

(5) $$\Delta a_{O_{2}}=a_{O_{2}ads}-a_{O_{2}des}$$

(6) $$\Delta a_{N_{2}}=a_{N_{2}ads}-a_{N_{2}des}$$

where $a_{i_{ads}}$ the adsorption bed capacity for the *i*-th gas at the adsorption conditions, $a_{i_{des}}$ is the adsorption bed capacity for the *i*-th gas at the desorption conditions.

The mass of oxygen $m_{O_{2}}$ separated in one adsorption cycle on one kg of the adsorption bed can be calculated using the equation:

(7) $$m_{O_{2}}=\left(\frac{0.22*\left(\Delta a_{N_{2}}\right)}{0.78}-\left(\Delta a_{O_{2}}\right)\right)*M_{O_{2}}$$

where $M_{O_{2}}$ is the oxygen molar mass.

**Figure 6:**
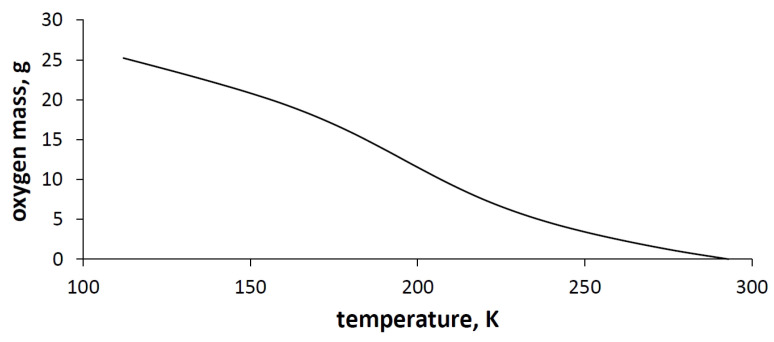
The oxygen mass obtained from the TSA method on 1 kg of adsorbent in one adsorption cycle.

The amount of oxygen, that can be obtained from one kilogram of the adsorbent in one adsorption cycle is shown in Figure 6.

Figure 6 shows clearly that the lower the temperature of the adsorption stage, the higher the capacity of the TSA oxygen separator.

## 4. Capability of LNG Exergy Recovery

LNG can be stored in different pressures that correspond to the specific temperature. Additionally, the regasification can be driven to the certain temperature of natural gas that is the most suited for the customer. The amount of heat *Q* that is needed to be transferred to natural gas in the regasification process depends on the temperature difference between the temperature of the regasified natural gas ($T_{2}$) and the starting temperature of LNG ($T_{1}$). The regasification heat *Q* is equal to the sum of the phase change at the starting LNG temperature and heat needed to increase the temperature of natural gas to the end temperature of the regasification process. It can be calculated as the difference of the enthalpy between the regasified natural gas and the LNG at the starting conditions using the equation:

(8) $$Q=H_{NG}\left(T_{2},P_{2}\right)-H_{LNG}\left(T_{1},P_{1}\right)$$

The heat *Q* necessary to vaporize and superheat natural gas can be obtained from the adsorption bed. Simultaneously, during the regasification of the LNG, the adsorption bed will be cooled down to the temperature of the regasified natural gas for the adsorption stage. Equation (9) can be used to calculate the mass of the adsorption bed that can be cooled down using 1 kg of the LNG depending on the temperature difference ΔT between the temperature of desorption—equal to the ambient temperature, and the temperature of adsorption—equal to the temperature of the end temperature of the reboiled natural gas ($T_{2}$).

(9) $$m_{ads}=\frac{Q}{C_{p}*\Delta T}$$

Equations (7)–(9) can be used to calculate the mass of the oxygen that can be separated from the air for 1 kg of regasified LNG. For simplicity, it is assumed that during the regasification process, pressure of the natural gas is constant and equal to the pressure of LNG.

**Figure 7:**
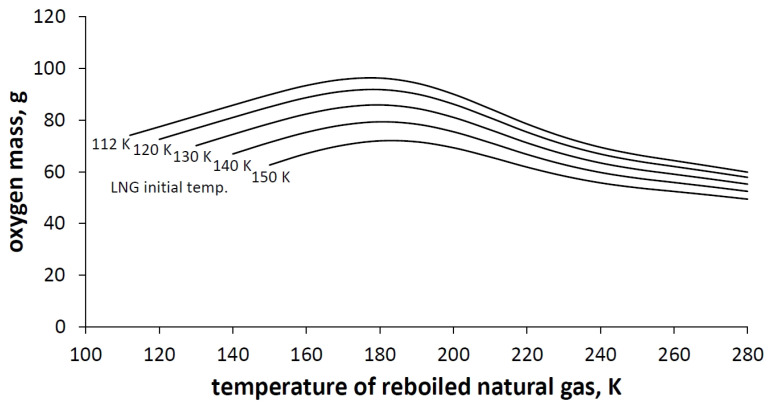
Grams of oxygen obtained from 1 kg of adsorbent in one adsorption cycle.

The amount of oxygen that can be obtained from the regasification of the unit mass of reboiled natural gas was calculated and is shown in Figure 7.

Figure 7 shows that the optimal temperature for the adsorption stage, which is equal to the end temperature of the natural gas, is equal to 180 K. Lower temperature results in too low a natural gas enthalpy difference and thus too low LNG cooling power. A higher temperature than 180 K results in a too low temperature difference between the adsorption and desorption stage and thus too low adsorption capacity of the one adsorption–desorption cycle. Table 5 shows the maximal calculated oxygen mass that can be obtained using the cooling power of LNG regasification.

Depending on the initial LNG storage conditions it is possible to separate up to 96.3 g of oxygen on 1 kg of adsorbent using the TSA method.

## 5. Conclusions

The TSA technology of air separation is effective and justified to get coupled with the LNG regasification units. The oxygen generation rate using the presented method depends on two temperature levels. First is the initial temperature of the LNG and the second is the final temperature of the regasified NG, which is equal to the temperature of adsorption. The analysis showed that in order to obtain the highest oxygen efficiency, the initial temperature of LNG should be as low as possible. In the case of the final temperature of regasified NG, there is an optimum allowing the maximization of oxygen production. The optimal final temperature of natural gas for the oxygen separation using the TSA method is 180 K. Lower temperature increases the adsorptive capacity of the adsorption bed but results in too low a natural gas enthalpy difference. A higher temperature than 180 K results in a low temperature difference between the adsorption and desorption stage and thus low adsorption capacity of the one adsorption–desorption cycle. It has been shown that 1 kg of LNG carries enough potential for separating up to approximately 100 g of oxygen (with a purity of 95%) depending on the temperature difference between NG and LNG. In other words, during a process of regasification of 1000 $m^{3}$ NG (expressed in normal conditions), it is possible to separate from air approximately 46 $m^{3}$ of oxygen with 95% purity using the 5A Zeolite adsorption bed and TSA method. It is worth noting that, because of the thermal inertia, all the processes of cooling and reheating of the adsorption bed could take a significant amount of time depending on the shape and volume of the adsorption tank. To obtain a continuous or semi-continuous separation process, several adsorption vessels would be needed.

## Figures and Tables

**Table 1:** Natural gas composition depending of the gas source [12,13].

Source	${\mathrm{CH}}_{4}$	$C_{2}H_{6}$	$C_{3}H_{8}$	$C_{4}H_{10}$	$C_{5}H_{12}$	${\mathrm{CO}}_{2}$	$N_{2}$	He
Algeria	86.9	9	2.6	1.2	-	-	0.3	-
Canada	71.8	-	13.9	13.94	-	0.3	12.4	0.6
India	73.2	6.1	3.2	1.6	0.6	0.3	14.3	0.7
Libya	70	15	9 3.5	1	-	2	-
Poland	89.9	1.6	0.9	0.7	-	0.3	6.5	-
Ukraine	97.8	0.5	0.2	0.1	0.05	0.05	1.3	-
USA (Alaska)	95.5	0.05	0.01	-	-	0.01	0.43	-
Venezuela	70.9	8.2	8.2	6.2	3.7	2.8	-	-

**Table 2:** Dual-Site Langmuir (DSL) isotherm coefficients for 5A Zeolite adsorbent.

Parameter	Nitrogen	Oxygen
$a_{mb},\mathrm{mol}/\mathrm{kg}$	0.7	
$K_{b}^{0},{\mathrm{bar}}^{-1}$	1.214	0.051
$Q_{b},J/\mathrm{mol}$	22,864.53	15,561.08
$a_{md},\mathrm{mol}/\mathrm{kg}$	3.2	
$K_{d}^{0},{\mathrm{bar}}^{-1}$	0.073	0.051
$Q_{d},J/\mathrm{mol}$	17,859.21	15,561.28

**Table 3:** Adsorption isotherm of pure nitrogen on zeolite 5—experimental data [19].

Temp. = 23 ${}^{{}^{\circ}}$C		Temp. = 45 ${}^{{}^{\circ}}$C	
Pressure	Degree of Adsorption	Pressure	Degree of Adsorption
kPa	mol/kg	kPa	mol/kg
8.6	0.0894	12.0	0.0669
26.1	0.2272	36.8	0.1816
54.2	0.3987	73.0	0.3193
121.2	0.6951	153.9	0.5584
233.5	1.0345	278.0	0.8346
388.2	1.3574	438.3	1.1013
640.1	1.7085	693.6	1.4117
967.0	2.0129	1020.3	1.6951
1341.5	2.2529	1393.6	1.9298
1741.0	2.4371	1790.9	2.1158

**Table 4:** Adsorption isotherm of pure oxygen on zeolite 5A—experimental data [19].

Temp. = 23 ${}^{{}^{\circ}}$C		Temp. = 45 ${}^{{}^{\circ}}$C	
Pressure	Degree of Adsorption	Pressure	Degree of Adsorption
kPa	mol/kg	kPa	mol/kg
20.3	0.0407	22.1	0.0281
47.6	0.0941	60.3	0.0767
96.3	0.1806	107.1	0.1344
194.5	0.3557	205.5	0.2484
329.3	0.5603	339.8	0.3954
534.0	0.8336	499.9	0.5553
786.3	1.1154	749.7	0.7773
1080.1	1.3861	1062.0	1.0157
1390.2	1.6213	1416.0	1.2443
1766.1	1.8436	1796.8	1.4499

**Table 5:** Maximal oxygen mass separated from one cycle for 1 kg of the reboiled Liquefied Natural Gas (LNG).

Initial LNG Temperature, *K*	Obtained Oxygen, $g_{O_{2}}/kg_{LNG}$
150	71.9
140	79.3
130	85.9
120	91.8
112	96.3

## References

[1] Hönig V., Prochazka P., Obergruber M., Smutka L., Kučerová V. (2019). Economic and Technological Analysis of Commercial LNG Production in the EU. Energies.

[2] Ren T., Patel M.K. (2009). Basic petrochemicals from natural gas, coal and biomass: Energy use and CO_2_ emissions. Resour. Conserv. Recycl..

[3] Agarwal R., Rainey T.J., Rahman S.M.A., Steinberg T., Perrons R.K., Brown R.J. (2017). LNG Regasification Terminals: The Role of Geography and Meteorology on Technology Choices. Energies.

[4] Vatani A., Mehrpooya M., Palizdar A. (2014). Advanced exergetic analysis of five natural gas liquefaction processes. Energy Convers. Manag..

[5] Zhang J., Meerman H., Benders R., Faaij A. (2020). Comprehensive review of current natural gas liquefaction processes on technical and economic performance. Appl. Therm. Eng..

[6] Chen Q.S., Wegrzyn J., Prasad V. (2004). Analysis of temperature and pressure changes in liquefied natural gas (LNG) cryogenic tanks. Cryogenics.

[7] Yang J.H., Yang G.S. (2014). The Temperature Field Research for Large LNG Cryogenic Storage Tank Wall. Appl. Mech. Mater..

[8] Messinero A., Panno G. (2011). LNG cold energy use in agro-food industry: A case study in Sicily. J. Nat. Gas Sci. Eng..

[9] Le S., Lee J.-Y., Chen C.-L. (2018). Waste cold energy recovery from liquefied natural gas (LNG) regasification including pressure and thermal energy. Energy.

[10] Gizicki W., Banaszkiewicz T., Wojcieszak P., Rogala Z. (2019). Performance analysis of small-scale power cycles for LNG physical exergy recovery. IOP Conf. Ser. Mater. Sci. Eng..

[11] Gómez M.R., Garcia R.F., Gómez J.R., Carril J.C. (2014). Review of thermal cycles exploiting the exergy of liquefied natural gas in the regasification process. Renew. Sustain. Energy Rev..

[12] Kordylewski W. (2005). Paliwa. Spalanie i Paliwa.

[13] Faramawy S., Zaki T., Sakr A.A.-E. (2016). Natural gas origin, composition, and processing: A review. J. Nat. Gas Sci. Eng..

[14] Włodek T. (2019). Analysis of boil-off rate problem in Liquefied Natural Gas (LNG) receiving terminals. IOP Conf. Ser. Earth Environ. Sci..

[15] Jee J.-G., Kim M.-B., Lee C. (2005). Three-bed PVSA process for high-purity O_2_ generation from ambient air. AIChE J..

[16] Lee S.-J., Jung J.-H., Moon J.-H., Jee J.-G., Lee C.-H. (2007). Parametric study of the three-bed pressure-vacuum swing adsorption process for high purity O_2_ generation from ambient air. Ind. Eng. Chem. Res..

[17] Hidano T., Nakamura M., Nakamura A., Kawai M. (2011). The downsizing of a TSA system for an air purification unit using a high flow rate method. Adsorption.

[18] Banaszkiewicz T., Chorowski M. (2018). Energy Consumption of Air-Separation Adsorption Methods. Entropy.

[19] Talu O., Li J., Kumar R., Mathias P.M., Moyer J.D., Shork J.M. (1996). Measurement and analysis of oxygen/nitrogen/5A-zeolyte adsorption equilibria for air separation. Gas Sep. Purif..

